# Perspectives From Multidisciplinary Professionals in France on Shared Patient Portals for Integrated Pediatric Rehabilitation: Qualitative Study

**DOI:** 10.2196/73068

**Published:** 2025-10-10

**Authors:** Marietta Kersalé, Quan Nha Hong, Thomas Richard, Christèle Kandalaft Cabrol, Audrey Guevel, Emmanuelle Fily, Gaëlle Tisserand, Sylvain Brochard, Marie-Pascale Pomey, Christelle Pons

**Affiliations:** 1 Paediatric Physical Medicine and Rehabilitation Department, CHU Brest Brest France; 2 Faculty of Medicine and Health Sciences University of Brest (UBO) Brest France; 3 Laboratory of Medical Information Processing (LaTIM), INSERM UMR 1101 Brest France; 4 W.Inn, Innovation Center, CHU Brest Brest France; 5 Faculty of Medicine University of Montreal Montreal, QC Canada; 6 Centre for Interdisciplinary Research in Rehabilitation of Greater Montreal (CRIR) Montreal, QC Canada; 7 University Paris-Est Créteil Créteil France; 8 Paediatric Physical Medicine and Rehabilitation Department, Fondation Ildys Brest France; 9 Research Center, University Hospital Center of the University of Montreal (CHUM) Montreal Canada; 10 Centre of Excellence on Patient and Public Partnership (CEPPP) Montreal, QC Canada

**Keywords:** children with disabilities, pediatric rehabilitation, integrated care, shared health patient portal, patient and family-centered care, patient and family partnership, education, recreation and community, social, qualitative study

## Abstract

**Background:**

Providing integrated care is essential in pediatric rehabilitation, as children with disabilities often navigate complex, long-term pathways involving multiple professionals across health, education, and community services. Strengthening communication and partnership among children, families, and professionals is key to supporting meaningful participation in daily life. Shared digital health portals offer a promising solution to support integrated care, yet their potential remains underexplored in this context.

**Objective:**

This study explores the perspectives of multidisciplinary professionals involved in pediatric rehabilitation on shared patient portals designed to support integrated care for children with disabilities.

**Methods:**

An interpretive descriptive qualitative study grounded in a constructivist epistemological position was conducted. Data were collected through semistructured online interviews with professionals purposively recruited using maximum variation sampling. All were involved in the rehabilitation pathways of children with motor, cognitive, or mental disabilities in France. Interview verbatim transcripts were analyzed using NVivo (version 14, Lumivero) by an interdisciplinary team of researchers, including parents and clinicians, using a thematic analysis approach. Theoretical saturation was reached.

**Results:**

A total of 32 professionals, including clinicians, educators, social workers, and coaches working in hospitals, rehabilitation centers, outpatient clinics, or private practices, integrated health and social services, schools, nurseries, leisure associations, and social services, participated in this study. Four themes captured professionals’ ambivalent perspectives on portals, addressing their perceived contributions to integrated care, anticipated barriers, practical strategies for implementation, and expectations regarding features: (1) ensuring continuity across health, education, and recreation services: navigating transparency, confidentiality, and inclusion; (2) enhancing family partnership while preserving professional autonomy and navigating engagement diversity; (3) involving children in patient portals: from children’s empowerment to professionals’ ethical responsibility; and (4) the contrast between concerns about additional workload and beliefs regarding efficiency. Participants suggested features such as shared calendars, secure messaging, and tools to share videos, rehabilitation goals, and track progress, alongside practical strategies to support real-world adoption.

**Conclusions:**

This study highlights the perceived potential of shared patient portals to strengthen partnerships and fruitful collaboration among children, families, and professionals involved in the pediatric rehabilitation pathways, including education and recreation providers. Professionals proposed concrete features to support integrated care, informing the development of tools likely to improve the quality of rehabilitation services. Future studies should explore the perspectives of children, families, and decision makers to support effective implementation and evaluate the real-world impact.

**Trial Registration:**

ClinicalTrials.gov NCT06570148; https://clinicaltrials.gov/study/NCT06570148

## Introduction

Children with disabilities face “long-term physical, mental, intellectual, or sensory impairments that, when interacting with various barriers, may hinder their full and effective participation in society on an equal basis with others” [1]*.* Pediatric rehabilitation aims to support meaningful participation in daily life, not only through health care but also by addressing educational, social, and community dimensions [2,3]. Rehabilitation in childhood is inherently interdisciplinary and family-centered, requiring active collaboration between families and a diverse range of professionals [4,5]. This broad perspective requires coordinated and continuous support over time, between visits and throughout the child’s development, including critical transitions into adulthood [6]. Therefore, effective communication and information sharing among all stakeholders are essential to ensure that care remains responsive to each child’s evolving needs and preferences [7] and are key to delivering integrated care, which is characterized by coordination, continuity, and shared decision-making with patients and caregivers [8,9].

Nevertheless, effective communication in pediatric rehabilitation remains a significant challenge [10]. Children with disabilities have long-term needs that involve multiple settings, organizations, and professionals [11], who rarely share information systems or communication standards. The dynamic nature of child development also creates challenges for maintaining continuity and adapting care over time. As a result, care pathways are often fragmented, interprofessional collaboration is limited [12,13], and families are left to navigate complex systems alone [14]. For professionals, poor care coordination leads to redundant efforts, inefficient communication, and increased workload [15]. These issues undermine care quality, contribute to poorer quality of life for children and families, and heighten the risk of unsuccessful interventions [10], resulting in higher service use [16].

Digital solutions, including shared patient portals, offer promising ways to improve communication and care integration [17,18]. These tools can facilitate access to information, support shared decision-making, and enable better coordination between children, families, and professionals [19,20]. While patient portals are increasingly recognized as valuable tools in other health care contexts [21], such as among older adults [22] and in stroke care [23], the potential for their use remains underdeveloped and underexplored in the context of childhood disability. To our knowledge, no existing portal has been specifically designed for pediatric rehabilitation, which involves complex, long-term, and cross-sectoral care requiring coordination across evolving developmental stages [11,24]. As a result, there is a need to develop more knowledge and tools specifically designed to meet the requirements of integrated pediatric care and that are better suited to real-world practice.

Professionals are both primary users and key gatekeepers of these solutions. However, studies show that professionals often perceive significant barriers to adopting eHealth tools, including concerns about workload, data privacy, and the usability or relevance of available solutions [25,26]. Therefore, to ensure that digital tools effectively support integrated pediatric rehabilitation and align with professionals’ practices, it is essential to gather their perspectives, including their concerns, needs, and expectations [27]. Taking this feedback into account and co-designing digital technologies with users ensures that they address real-world needs and challenges [28].

This study aims to address this gap by exploring the perspectives of professionals involved in pediatric rehabilitation regarding shared digital health portals designed to support integrated care for children with disabilities.

The following research questions guide the research:

- How do professionals perceive the potential contributions of shared digital health portals to integrated pediatric rehabilitation care?
- What barriers and concerns do they anticipate in using such portals in their practice?
- What practical solutions do professionals identify to support successful implementation in real-world settings?
- What are their expectations regarding key portal features to support integrated pediatric rehabilitation care?

This study is part of a broader research project exploring the perspectives of children with disabilities, their families, and professionals on shared digital health portals for integrated pediatric rehabilitation. As professionals play a critical role in implementing and supporting such tools, understanding their perceived usefulness, concerns, and constraints is essential for designing a portal that can meaningfully support integrated rehabilitation care for children and families. These findings will directly inform upcoming participatory phases involving children and families to co-design a portal that meets stakeholders’ needs and can be realistically adopted in real-world settings.

## Methods

### Study Design

An interpretive descriptive qualitative methodology was used [29], as it offered an appropriate approach to explore complex experiential questions while producing practical outcomes [30]. We adopted a constructivist epistemological position, recognizing that reality is multiple, contextual, and socially constructed [31], to generate meaning through a collaborative dialogue between researchers and participants.

Semistructured individual interviews were conducted to explore professionals’ thoughts, attitudes, and perceptions in depth [32]. An interview guide was designed by authors MK and CP, based on the research questions, with feedback from the researchers. The guide was pretested with a physiotherapist who had experience in different pediatric rehabilitation settings (hospitals, rehabilitation centers, and outpatient clinics or private practice) and modified throughout the interview process by the 2 interviewers (MK and TR). The final version of the guide contained 9 open-ended questions with prompts (Multimedia Appendix 1). The first part focused on the opportunities and concerns perceived by professionals about the role of **eH**ealth in facilitating the provision of integrated care in pediatric rehabilitation and suggested features to improve the pediatric rehabilitation pathway. In the second part, features of an existing health portal developed for adult rehabilitation (Deneo santé, Deneo SAS) were shown to the participants for (1) the sharing of reports, images, photos, and videos between patients and professionals, (2) a database of validated rehabilitation measurement scales and questionnaires, (3) the monitoring patient progress using graphs, and (4) the prescription of self-rehabilitation exercises. This demonstration aimed to help professionals visualize a concrete digital tool, explore the acceptance of using such a portal [33], and generate ideas for its features. Finally, participants were invited to recommend strategies for implementing such tools in their practice.

### Study Participants and Recruitment

The inclusion criteria were professionals involved in the rehabilitation pathway of children with physical, cognitive, or mental disabilities working in France. Purposeful sampling with maximum variation [34] ensured participant heterogeneity based on work setting, profession, age, years of experience, digital literacy, and region of France. Regional variation was considered to account for differences in pediatric rehabilitation care coordination across France. First, participants were selected according to these criteria through FRISBEE (Fédération Médicale et Universitaire des SMR Pédiatriques de Bretagne Occidentale) [35], a network coordinating research, health care, and teaching in pediatric rehabilitation in France. Snowball sampling [36] was also used to recruit additional participants, particularly from the education sector. More specifically, the first 5 participants interviewed highlighted the need to involve school assistants and professionals from leisure and sports associations in shared patient portals and provided contact details for relevant professionals. For this reason, from an integrated care perspective, both professionals working in rehabilitation settings (eg, physiatrists, occupational therapists, physiotherapists, kinesiologists, psychologists, speech therapists, nurses, and educators working in hospitals, rehabilitation centers, outpatient clinics or private practice, and integrated health and social services) and those in education and recreation settings (eg, nurses, psychologists, teachers, sports coaches, and those working in elementary, middle, and high school and sports and leisure associations) were included. MK and CP discussed each participant’s profile to ensure it differed from previous participants. Theoretical saturation was applied pragmatically to determine the final sample size [37], aligning with the constructivist epistemology of interpretive description, emphasizing practical, context-sensitive understanding rather than exhaustive thematic saturation.

### Data Collection

Interviews were conducted in French via videoconference (Zoom Pro, Zoom Video Communications, Inc) between February and July 2024. They were audio-recorded and lasted between 36 and 88 (mean 60, SD 9.9) minutes. Two researchers conducted the interviews: MK, an occupational therapist trained in France and Canada, PhD candidate in rehabilitation sciences and experienced in qualitative health research, and TR, an occupational therapist pursuing a Master’s degree in research. Both had received specific training in qualitative interviewing methods, including formal coursework and supervised practice. Neither researcher had any prior relationship with the participants. After each interview, they discussed the process to ensure consistency in the questions and to adjust the guide if needed, and took reflective notes to document their postinterview impressions and any issues for consideration in the analysis. Participants’ sociodemographic data were collected at the end of the interview (Table 1). Digital literacy was self-assessed using a Likert scale between 0=no competence and 10=full competence. A score of 10 meant being able to download an application, share files easily, and take ownership of a new technology. eHealth use was defined as using apps, web platforms, or health portals (excluding electronic health records) to communicate with providers or monitor children’s progress.

**Table 1:** Sociodemographic characteristics of participants.

Characteristics					Values
Gender, n (%)					
	Women			24 (75)	
	Men			8 (25)	
Age (years)					
	Mean (SD)			39.2 (8.1)	
	Range			26-55	
French region, n (%)					
	Brittany			11 (34)	
	Paris region			6 (19)	
	Auvergne-Rhône-Alpes			5 (16)	
	Pays de La Loire			2 (6)	
	Occitanie			2 (3)	
	Normandy			1 (3)	
	Nouvelle Aquitaine			1 (3)	
	Provence Alpes Côtes d’Azur			1 (3)	
	Bourgogne Franche Comté			1 (3)	
	Réunion Island			1 (3)	
	Grand Est			1 (3)	
Profession^a^					
	Rehabilitation professionals			14	
		Coordinator of a mobile team	1		
		Occupational therapist	3		
		Physiotherapist	3		
		Kinesiologist	3		
		Psychomotor therapist	2		
	Physicians			4	
		Physiatrist	2		
		Pediatrician	1		
		School physician	1		
	Psychologists			3	
		Psychologist	2		
		Neuropsychologist	1		
	Educators and educational assistants			4	
		Educators	2		
		Educational assistant	1		
		Childcare assistant	1		
	Teachers			3	
		Specialized teacher	1		
		Referent teacher	1		
		Coordinating teacher	1		
	Social workers			2	
		Social worker	1		
		Nurse	1		
	Sports coach			1	
	Team manager			5	
Type of working facility^a^					
	Hospital, n (%)			6 (19)	
	Rehabilitation center, n (%)			5 (16)	
	Integrated health and social services, n (%)			5 (16)	
	Outpatient or private practice, n (%)			4 (13)	
	Schools or nurseries			12 (38)^b^	
		Elementary school	3		
		Middle school	4		
		High school	1		
		All classes	2		
		Nursery	2		
	Leisure or sports associations			5 (16)^b^	
		Sport association	3		
		Leisure association	2		
	Social administration, n (%)			1 (3)	
Years of experience in pediatrics					
	Mean (SD)			12.9 (8.4)	
	Range			1-34	
Pediatric specialty, n (%)					
	General (any type of disability)			22 (69)	
	Specific (particular disability)			10 (31)	
Digital literacy (self-rated 0-10)					
	Mean (SD)			7.4 (1)	
	Range			4-10	
Previous or current use of shared patient portal (excluding clinician-only electronic health record), n (%)					
	No			25 (75)	
	Yes			7 (25)	

^a^Participants could have multiple professions and work in multiple types of facilities.

^b^n (%).

### Data Analysis

**Figure 1:**
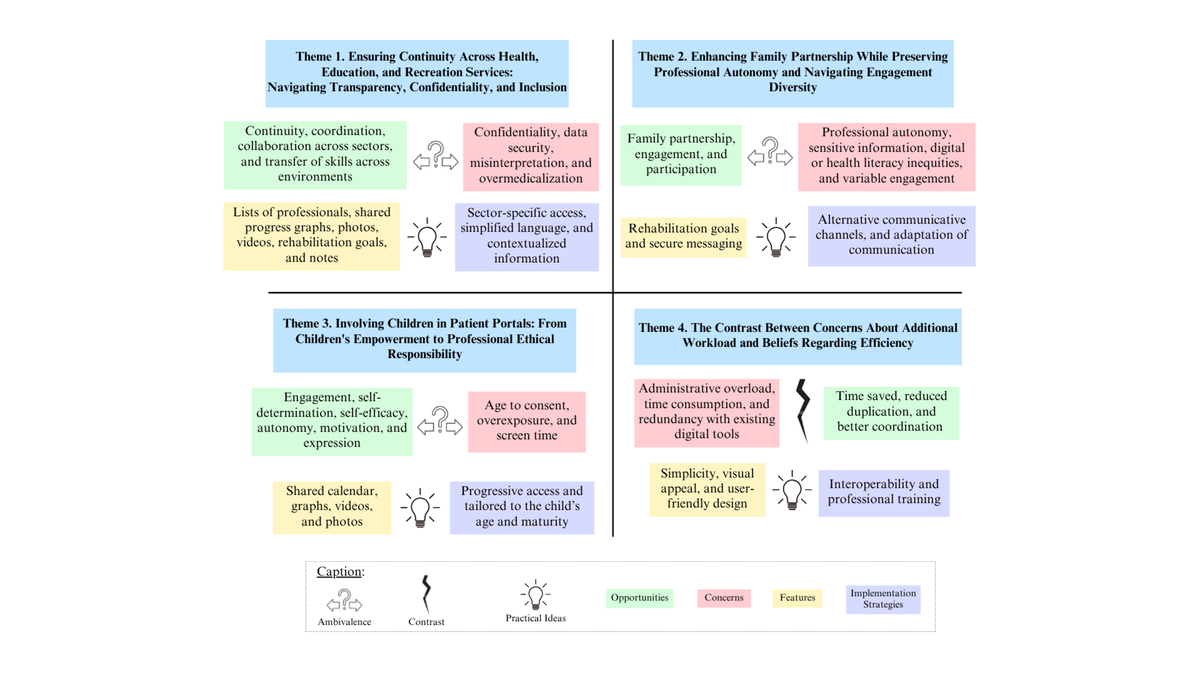
Thematic summary organized by the 4 research questions guiding this study (opportunities, concerns, desired features, and implementation strategies).

Audio recordings were transcribed verbatim using artificial intelligence–based transcription software (Easy-Peasy.AI, Easy-Peasy.AI Pte Ltd) and double-checked by the interviewers for accuracy. Thematic analysis was conducted following the 6-step approach by Braun and Clarke (2006) [38]. First, 2 researchers (TR and GT) independently coded 4 interviews using NVivo (version 14), based on an initial coding tree informed by the interview guide and aligned with the research questions (see colored squares in Figure 1). The aim of this initial double coding was not to compare or validate interpretations, but to generate a broad range of codes and foster analytical and reflexive dialogue that enriches the interpretive process. The resulting codes and subcodes were discussed with MK to refine the coding framework inductively, grounded in the data. The remaining interviews were then coded individually by TR, GT, and MK. Weekly meetings with CP supported the iterative refinement of themes. Themes identified by the coding team were based on patterns across the data and refined with input from the broader research team. Two parent partners (CKC and AG) critically reviewed transcripts and theme labels to ensure balanced interpretation of the diverse professionals’ perspectives and supported reflexivity by questioning assumptions embedded in the data. Their input contributed directly to the refinement of cross-setting themes, such as the ambivalence within the principles of family and patient partnership. MK and CP reviewed and renamed the themes and subthemes during this paper’s preparation.

Data saturation was assessed as a practical and iterative judgment during data collection and analysis [39]. After each interview, the coders discussed emerging codes with CP to determine whether additional data contributed substantively new insights to the evolving interpretation [37]. After 30 interviews, no new codes or themes emerged; new data only confirmed or deepened existing themes. To confirm saturation, 2 additional interviews were conducted with participants from slightly different professional backgrounds (P31 and P32, see Table 1).

### Trustworthiness and Reflexivity

To ensure trustworthiness, several strategies were adopted [31]. Credibility was supported by analyst triangulation involving researchers from diverse backgrounds (public health, physiatry, occupational therapy, and parent partners) [40]. Initial coding was performed independently by 2 researchers, followed by regular team discussions. Participants were invited to review their transcripts (5 accepted but none provided feedback), and an online member-checking discussion with 3 participants confirmed the findings’ accuracy [41]. Transferability was enhanced with detailed sociodemographic reporting to support contextual interpretation and the contextualization of findings in the discussion, thereby aiding readers in assessing their applicability to other settings. Dependability was addressed through a reflexive journal documenting methodological decisions. The COREQ (Consolidated Criteria for Reporting Qualitative Research) checklist was used to report the methodology of the current study and ensure that information was complete [42] (Multimedia Appendix 2). For confirmability, analysts individually responded to the reflexive question from the triangulated inquiry framework by Patton [43] to examine how personal and professional experiences influenced data collection and analysis, and they shared their reflections during team meetings to foster critical reflexivity [44]. For example, the principal investigator (MK) acknowledged that her strong commitment to patient partnership initially led her to emphasize data supporting this perspective. Through ongoing reflexive discussions within the team, particularly with parent partners, she critically re-evaluated her decisions to ensure a more balanced representation of diverse and contrasting viewpoints.

### Ethical Considerations

This study was approved by the “Comité de Réflexion Éthique de Territoire”—Territorial Ethics Reflection Committee—of Brest University Hospital (B2024CE.05). All participants received an information and consent form and consented to the audio recording. Each participant was assigned a pseudonym, and any identifying information was removed from the verbatim transcriptions of interviews. The names of the children mentioned in the verbatim were changed. Participants did not receive financial compensation for their participation.

## Results

### Participants’ Profiles

In total, 45 professionals were contacted by phone, email, or LinkedIn. Ten professionals did not respond, and 3 refused because they considered themselves inexperienced (n=2) or had recently changed jobs (n=1). The final sample consisted of 32 professionals, with 20 different professions represented, working in a hospital (n=6), rehabilitation center (n=5), outpatient clinic or private practice (n=4), integrated health and social services (n=5), high school (n=1), middle school (n=4), elementary school (n=3), nursery (n=2), sports associations (n=3), leisure associations (n=2), and social administration (n=1). Four professionals were working in 2 different settings. The median age of the participants was 39 (range 26 to 55, IQR 32.8-46.3) years, with a majority of women (n=24), and they came from 11 regions of France (out of 13). The median digital literacy score was 7.4/10 (range 4 to 10, IQR 7.0-8.0). Seven participants have already used shared patient portals. Participants’ characteristics are described in Table 1.

### Specific Results

#### Overview

The analysis revealed 4 themes related to the perspectives of professionals on digital health portals in pediatric rehabilitation: (1) ensuring continuity across health, education, and recreation services: navigating transparency, confidentiality, and inclusion, (2) enhancing family partnership while maintaining professional autonomy and navigating engagement diversity, (3) involving children in patient portals: from children’s empowerment to professionals’ ethical responsibility, and (4) the contrast between concerns about additional workload and beliefs regarding efficiency. The 4 themes are summarized in Figure 1, shown as colored squares linked to the 4 research questions on portal contributions, barriers, solutions, and key features. Colors represent different aspects of professionals’ perspectives: green for opportunities, rose for concerns, yellow for features, and violet for implementation strategies.

#### Theme 1: Ensuring Continuity Across Health, Education, and Recreation Services: Navigating Transparency, Confidentiality, and Inclusion

All participants perceived shared patient portals as a meaningful opportunity to build a rehabilitation continuum, bridging existing gaps in collaboration between health care, education, and recreation services. For education professionals, this fragmentation was often attributed to differing professional cultures and practices:

[Health-school] meetings [once a year] are a battle to know who [health professional or teacher] is going to succeed in doing something. We're too much in opposition. We don't work together. There's a school-care divide.

P21, psychologist in an elementary school

The rehabilitation professional often works individually and observes things about behavior in a dual relationship. But these observations are not necessarily the same in a group setting like the classroom. It needs to be refined so that these two worlds, which work in completely different ways, can agree on each other's needs.

P17, referent teacher in an elementary school

To address this, some participants suggested identifying and clearly displaying all professionals involved in a child’s care through a list, for example, to facilitate communication and collaboration:

If [with the app] we can link the school with rehabilitation and outpatient care. It would change our lives, that's for sure.

P08, occupational therapist in health and social services

A list and photos with regular monitoring, other professionals involved in the past, present, and future.

P12, specialized teacher in an elementary school

A shared portal was envisioned by professionals in the community as a tool to bring professionals together around the child and place them on equal footing, recognizing the valuable insights of education and recreation professionals:

For educational continuity [...] without splitting up the child and their pathway. [...] An educator in a leisure setting provides information just as important because it's a social space that differs from home or school. [...] It has to be put on the same level to create richness for individual and group observations.

P19, educator and manager in a leisure association

Being all together on one platform puts everyone on an equal footing [...] It could allow people to feel like they're functioning together in the same direction for the child.

P21, psychologist in an elementary school

To support this continuity, some participants suggested that shared progress graphs could strengthen the school’s sense of involvement in the child’s developmental journey:

Highlighting the child’s shared progress would help the school feel more connected with the collective success.

P21, psychologist in an elementary school

Almost all professionals emphasized the importance of exchanging information updates about the child’s development across different environments, considering the evolving needs of children with disabilities. They highlighted the value of sharing practical information about adaptations and tools familiar to the child in everyday contexts to support children’s participation, through shared notes or secure messaging:

For young, autistic children in the early stages of the diagnosis, there is a very frequent need for information from the parents on all the stages of development, the children's progress, at home, at school, at the nursery, in the playground...

P28, neuropsychologist in health and social services

A chat, with one-on-one or group conversations, would be amazing. We could easily communicate with each other about the child.

P03, psychomotor therapist in private practice

Health care professionals valued the importance of sharing these daily observations with education professionals to help them understand how the child applies new skills across settings:

Something like a life notebook at school, to share everything the child does daily between partners, to be able to generalize all their learning.

P28, neuropsychologist in health and social services

To be able to exchange [with school] on useful adaptations [...] alternative and augmentative communication tools [...] how to avoid stimuli that provoke a crisis [...] small things that we can all easily implement and that can be very, very facilitating for children.

P15, speech therapist in health and social services

To support this transfer of skills, some professionals also proposed sharing rehabilitation goals through visual content such as photos, videos, or daily observations, making it easier for other stakeholders to reinforce these skills in everyday situations:

Knowing what the goals of rehabilitation, medical follow-up, or school would allow us to say “perhaps we can do the same.”

P23, manager and kinesiologist in a leisure association

Tying shoelaces is something we can do […] With a short video, we would know how to explain it to him.

P26, childcare assistant in a nursery

However, some participants warned of the risks associated with misinterpretation, especially when information is presented without proper context:

Control the dissemination of data to avoid interpretations that could be prejudicial to young people. [...] If you take an event out of its context and the whole history of the young person, it can be dangerous, because you can stick a label on a young person.

P31, coordinating teacher in a middle school

Therefore, a professional emphasized the need for systematically contextualized and nuanced information within portals to avoid reductive or inaccurate conclusions:

From one environment to another, children’s reactions are going to be different. So, we can't restrict ourselves to the platform and information from other professionals.

P23, manager and kinesiologist in a leisure association

One participant explicitly opposed sharing information with professionals from the education sector, citing strong concerns about confidentiality:

In healthcare, there's a certain level of security, but when you mix with school... During team meetings with the school, we don't talk much about medical information in front of educators and teachers. So, there's a concern about data protection when you involve many people from different backgrounds.

P24, physiotherapist in an outpatient clinic

To mitigate these concerns (Textbox 1), several participants proposed sector-specific access to shared portals, including dedicated sections and simplified language for non–health care professionals:

We could imagine specific access for teachers [...] I used to write my reports so that they could be read by the teachers, always, always, always. So that this exchange could take place.

P16, speech therapist in a hospital

**A large majority of participants emphasized that improved information exchange could enhance children’s participation in daily life contexts, such as home, school, and recreation. This continuity was seen as essential for children’s inclusion. These are the quotes on this.**

- A child with a motor disability can still participate in certain activities. I think this could reassure teachers about what they can expect from a pupil. Because they might be afraid of doing something wrong.
   [P20, social worker in a middle school]
- At school, after 3 minutes, Sacha was red-faced and couldn't take it anymore. So, they'd quickly get worried and put him aside. As a result, he didn’t do any sports at school. It would have been a good idea to tell them no, he can do it, to show them Sacha's limits, they would have known what's possible for him.
   [P30, coach in a sports association]
- To have a real continuity. Learning happens through repetition, and a one-hour-a-week session isn't enough [...] If on the app we can see where the child is at and say to parents: “he's capable of doing this, this week, you can encourage him to do it.” [...] About the child's skills during the session and how we can transfer them to the classroom.
  [P08, occupational therapist in health and social services]

However, one professional warned that shared portals could reinforce a specialized, overmedicalized framing of disability, blurring the line between inclusive practices and rehabilitative logics:

The kid already makes the effort to go to physio, OT… and now, they’ll go to leisure activities the same way, as if it’s just another rehab session. […] We have to be careful not to fall back into a logic where everything gets over-medicalized. Some families fought hard to get out of the specialized system. […] Even if the intention is inclusive, the tool could make it feel like we’re going backwards.

P19, educator and manager in a leisure association

#### Theme 2: Enhancing Family Partnership While Preserving Professional Autonomy and Navigating Engagement Diversity

All participants mentioned communication gaps with families, and some professionals emphasized that this lack of coordination and communication compromised family engagement and continuity of care:

When there's a lack of communication between the different partners, the family might not adhere so well. [P31, coordinating teacher in a middle school]

Portals were perceived by several participants as a potential tool to strengthen partnerships with families by facilitating sustained contact and transparency, which they felt was essential to effective rehabilitation:

An app would be great if it helped foster links between professionals and families.

P15, speech therapist in health and social services

[in rehabilitation] we have a different approach with children and families, considering them as partners, with a more inclusive approach, with the ultimate aim of achieving self-determination in adulthood. e-health has an important role in this follow-up and the participation of patients and families. [P13, physiatrist in a hospital]

More specifically, 1 participant mentioned that portals could support active family involvement in setting and tracking rehabilitation goals, which could, in turn, foster greater family participation:

Seeing that they are the ones who set the goals, and the goals are worked on by all stakeholders, through something coordinated, families would be more willing to participate in rehabilitation.

P01, coordinator of a mobile rehabilitation team

A few professionals suggested integrating secure messaging for families, but with clear boundaries, to prevent overuse or intrusion:

A messaging system with families. But the framework would need to be defined with them to define boundaries. It shouldn’t become overwhelming.

P02, psychologist in a rehabilitation center and private practice

However, participants expressed divergent views about the degree and nature of family involvement. Several professionals raised concerns about how parental access might limit open communication between colleagues, particularly when navigating complex or sensitive issues:

When children have behavioral or psychological difficulties, it's not easy. The child's legal representatives shouldn't have access because it's complicated. Sometimes, it's hard to talk about certain things.

P16, speech therapist in a hospital

There's information that I wouldn't want to tell the family, but that I would want to tell other professionals.

P03, psychomotor therapist in private practice

Sharing emotions between professionals is part of our job, and the parents should not necessarily take part in that…

P06, occupational therapist in a hospital

To mitigate this, 1 participant advocated full transparency with families but stressed the need for parallel channels to handle sensitive matters:

I wouldn't feel comfortable putting up documents that parents couldn't access. [...] For me, parents are 100% responsible for their child's care.

...] I'm not going to share concerns about abuse on this app for the attending physician to see. I would drop in or make a phone call. It would be better for parents to keep control of the app. [P28, neuropsychologist in health and social services

Other participants emphasized that if families were granted access, the information shared should be accessible and understandable to them, requiring professionals to modify how they communicate:

If they have access, the idea is for patients and parents to be partners. After that, it's up to the professionals to adapt, to share the difficulties they encounter with patients and parents, and to use words that everyone can understand.

P06, occupational therapist in a hospital

Still with an adapted language, several participants underlined structural barriers to family engagement in digital platforms, including digital literacy, language, and social precarity, which could exacerbate inequalities in access and engagement:

For foreign parents or those living in poverty, written information can be very difficult to access.

P27, educator in a rehabilitation center

In our area, we have quite a few illiterate parents, so that would be complicated.

P13, physiatrist in a hospital

Still, not all professionals believed that digital tools would necessarily foster stronger engagement. One participant expressed frustration with what they perceived as persistent family disengagement:

Sometimes, families just don’t care at all. Depending on the families, but some don’t help us.

P24, physiotherapist in an outpatient clinic

#### Theme 3: Involving Children in Patient Portals: From Children's Empowerment to Professional Ethical Responsibility

Participants highlighted the potential of patient portals to engage adolescents in their rehabilitation journey meaningfully. All participants mentioned at least 1 way these tools could empower children by increasing transparency around their health data. Given the long duration of rehabilitation and the potential fatigue children experience, many professionals viewed portals as a valuable tool to support self-awareness, particularly during adolescence, with features to visualize their rehabilitation goals and track their progress over time:

In high school, rehabilitation follow-up is often more difficult because it's the teenage period. Young people are a little fed up with rehabilitation. [The app] would give them a global vision of what it's all about, what the purpose is, and everything they've achieved.

P20, social worker in a middle school

Children could see their progress, with different levels along the way. If the goal is to ride a two-wheeled bike to school, and they’re able to keep their balance on the way home, that’s already great. It could encourage the child.

P06, occupational therapist in a hospital

Most professionals also emphasized how patient portals could support clearer communication for children, helping them understand their rehabilitation journey and the purpose behind different interventions, through a unified discourse:

Children have a lot of different people working with them, and as it's always a dual relationship, if no one has a somewhat uniform discourse, it creates vagueness, and I think it's deleterious for the child.

P29, physician in a middle school

The coordination provided by the app would make it possible to have coherent and consistent care for children. They would see that everyone is working towards the same goal, and that there isn't one professional doing one thing, and another doing something else, and another different technique at school.

P01, coordinator of a mobile rehabilitation team

Maintaining motivation was another key opportunity. By allowing children to track their progress with visual tools such as graphs, videos, or photos, some professionals believed that portals could enhance self-efficacy and motivation, particularly for children with disabilities who do not realize their potential:

A lot of kids doubt their skills. [...] Having graphs could help them to see their progress from the beginning and see that they succeeded quite well in the exercises.

P16, speech therapist in a hospital

Being able to validate children's progress through videos would be motivating for the child. I am sometimes quite surprised by how a child does not realize the extent of their skills.

P08, occupational therapist in health and social services

One professional perceived patient portals as a way to address the communication challenges faced by some adolescents, especially those who struggle to express themselves during consultations:

A digital tool, especially for teenagers, who don't necessarily talk during consultations because their parents are present. [...] It has to be the child who fills in the application so that they’re invested in it. That they have access to their data and distribute it to whoever they wish.

P25, physiatrist in a rehabilitation center

Several participants also recognized the importance of treating children as partners in their care, just as they did with families. This partnership dynamic was emphasized as a key advantage of portals:

The relevance of this tool compared to others would be the patient partnership. It's not a transmission software between professionals; it's rather a tool that enables collaboration with patients. That would be the real added value.

P02, psychologist in a rehabilitation center and private practice

Despite these promising aspects, several professionals expressed several concerns related to their ethical responsibility to protect children. Notably, there was a divergent view about the appropriate age for children to consent, with participants suggesting a wide range of ages. One key concern was granting children early access to health data, which they feared could complicate communication in sensitive situations:

We already use filters with parents, but if the child has access, we’ll filter even more. […] There are meetings in which the situation is very complicated, but we don't want to hurt the parents, so nothing is said during the meeting. And then when we come out, no one is happy because we didn't address the real problems, and we didn't want to get in the way. So, I think it’s better if the child is not in the app too early.

P29, physician in a middle school

Several participants also expressed concerns about the evolving nature of children’s consent and the possibility that they might later regret having shared certain information:

There are perhaps not the same commitments in terms of consent when you are 6 years old or when you are 17.

P19, educator and manager in a leisure association

[For patients] not realizing what they are sharing and then regretting it, that’s what scares me a little.

P04, physiotherapist in health and social services

To address these concerns, some professionals suggested that access to such portals should be progressive, tailored to the child’s age and maturity. Early access could be limited to basic, visual elements, with more detailed features becoming available as children grow and develop autonomy:

It depends on each child’s maturity and needs. At six, maybe they can just see photos of who’s involved in their care, their role, the times or days. And then the rest can come gradually.

P12, specialized teacher in an elementary school

At first, they could use it with their parents. By around age 10, depending on maturity, they’re usually able to say what they need, how it works, and whether it’s helpful or not. They’re beginning to show the ability to reflect and make informed judgments.

P21, psychologist in an elementary school

Even with gradual access, some professionals remained concerned about excessive monitoring of children through portals, which could compromise their privacy and autonomy:

Not everyone should know everything. We are in a bit of a culture today of sharing everything, and I think we have to be wary because it is good for a young person to go to a place where not everyone knows their whole life story and their whole disability.

P14, community manager in a sports association

We must give others the freedom to be and to do what they want, and not to be tracked in terms of behavior or events. I would freak out if I had information about me saying that I was at the leisure center this summer, I was nice, and then afterwards, I got a little angry. That's life! [...] There's a red flag on that.

P19, educator and manager in a leisure association

Beyond age-related access concerns, 1 participant worried that full access to information might be too confronting for young people with severe or progressive disabilities:

It’s complicated because these children often mourn a professional project incompatible with their condition. Having access to all exchanges might add another burden when they already have a lot to bear. It should be evaluated case by case, considering the young person’s wishes and pathology.

P29, physician in a middle school

Finally, screen time was also raised as a concern by several participants, especially regarding its impact on children’s health and development:

We are already trying to tell parents to reduce screen time so much! If we offer videos or things on screen to children who are non-readers, I think it will have to be done well.

P15, speech therapist in health and social services

We try to get them off the screens, and then we give them an app! But at the same time, it’s also a tool that can be used positively, so it’s both.

P10, kinesiologist in a hospital

#### Theme 4: Contrast Between Concerns About Additional Workload and Beliefs Regarding Efficiency

Almost all professionals mentioned the additional time burden posed by digital portals as a primary concern. One of the main reasons was the presence of multiple existing technologies, especially when professionals had to enter information into various systems, such as electronic medical records:

We're already swamped with paperwork and things to do. At work, they're already asking me to fill out more and more documents. If, on top of that, I have an app where I have to fill in even more, that would be a huge hindrance for me.

P24, physiotherapist in an outpatient clinic

The barrier is the time it takes to use it. Because the way things are going, we are using digital tools more and more, and that doesn't always save time [laughs].

P29, physician in a middle school

To overcome workload challenges, many participants stressed the critical need for interoperability, emphasizing that portals must integrate smoothly with existing digital systems and allow flexible, privacy-compliant data sharing:

It should be connected to the hospital’s existing software, so that appointments scheduled can be automatically added to the shared calendar.

P06, occupational therapist in a hospital

It has to be developed in connection with the software used in the center. And for data sharing, ideally, there should be clear boxes you can tick to choose whether or not to share information.

P27, educator in a rehabilitation center

In addition to technical compatibility, some participants emphasized the importance of a simple, engaging, and visually appealing design to ensure adoption across diverse users. They advocated for an intuitive interface that avoids overwhelming users with too much information and instead allows such tools to evolve gradually, adapting progressively to users’ needs and digital familiarity:

It needs to be fun, visual, with appealing images. […] Not too much information, so people don’t get lost. The initial interface should be extremely easy to navigate, and then the platform can evolve gradually over time.

P07, kinesiologist in a sports association

By contrast, a few professionals viewed portals as a potential time-saving investment that could reduce repetitive communication and improve coordination:

I spend a lot of time and energy communicating the same information to everyone, and I find that quite exhausting. […] It would save time, because we’ll get quick answers instead of making a phone call or send an email and wait for a reply.

P01, coordinator of a mobile rehabilitation team

I'm rubbish at computers. I'd say to myself, “Oh no, another tool!” [laughs]. It probably wouldn't be easy to use at first, and I'd have to learn to use it, but afterwards I'd save time. And there would be a more fluid link.

P08, occupational therapist in health and social services

Importantly, divergent perceptions did not appear to be linked to professionals’ digital literacy, but rather to differing professional values and beliefs about coordination and the role of information sharing in care, as said by 1 professional:

It's typically the kind of platform where people believe that the more information we have and cross-reference, the better we manage to take care of the child. Some will see it as a kind of burden or extra work, and others will say it's a great tool and part of the job of taking care of young people.

P31, coordinating teacher in a middle school

To address these divergent perceptions, a few professionals proposed strategies, including training professionals and demonstrating the benefits of shared portals:

It’s important to show them how the tool will help, focusing on time saving and improving daily practice. And how it will contribute to the student’s success. Because that’s why we’re all here.

P05, nurse in a high school

## Discussion

### Principal Results

#### Overview

To our knowledge, this study is the first to focus on **eH**ealth to support integrated pediatric rehabilitation from the perspectives of health care, education, and recreation professionals. The findings revealed significant opportunities for shared patient portals to improve care continuity in rehabilitation by facilitating communication about children’s participation across different environments and fostering ongoing engagement with children and families throughout the rehabilitation process. Several features were proposed, including a list of involved professionals, a shared calendar across services and providers, the ability to share photos, videos, and reports of the child’s functioning in different environments, and the option to share progress with children and families. While many professionals perceived significant opportunities for improving care continuity and engagement through shared portals, divergent and at times contradictory views also emerged regarding confidentiality, collaboration across sectors, and the role of children and families in such systems. To ensure successful adoption, several strategies were suggested, including sector-specific access, adapted language, contextualized information, progressive access for children, system interoperability, and professional training.

#### Toward a Rehabilitation Continuum: From Greater Transparency to Fears About Confidentiality

Many professionals perceived shared portals as essential communication tools to ensure continuity across health care, education, and recreation services—a key determinant of integrated rehabilitation care [9]. This aligns with the need to redesign systems for children with disabilities using a systemic approach that addresses all health determinants, not just health care services [45]. Cross-sectoral collaboration is critical in pediatric rehabilitation, where providers and parents must work together across various environments to promote children’s development and participation [12,46,47]. The findings suggest features to integrate, such as a shared calendar, goals, and options for sharing observations and monitoring children’s progress through photos or videos. By providing structured and transparent communication, such features could support professionals in aligning their interventions and ultimately enhancing children’s abilities to participate successfully in school [3]. Yet, a minority of professionals opposed granting educational staff access to portal data, citing confidentiality and contextual misinterpretation concerns. Beyond education, some professionals emphasized the need to collaborate with professionals from sports and leisure associations to foster inclusion in leisure activities, collaborations that are currently lacking. Despite its importance, this cross-sectoral coordination remains underexplored in the literature, especially regarding recreation and education professionals, who are often overlooked as key stakeholders [48]. By enabling real-time information sharing, patient portals could provide a more holistic understanding of children’s needs and participation across different environments, supporting tailored interventions and aligning with the “F-word” framework, which highlights 6 key aspects of childhood disability: Functioning, Family, Fitness, Fun, Friends, and Future [49].

However, participants reported several barriers to cross-sectoral collaboration, particularly between professionals working in rehabilitation and education settings, including divergent work methods and approaches, which represent a significant challenge to inclusive schooling [50]. Although concerns about confidentiality are consistent with previous studies [20,51], deeper tensions emerged regarding giving non–health care professionals access to clinical observations in shared portals. In France, education professionals are bound by professional secrecy [52], which means they cannot access health data unless explicitly authorized by a major patient or legal representative, but they are authorized to have access to observations about children. These legal distinctions are reinforced by the European General Data Protection Regulation [53], which emphasizes the right of patients, or for minors, their legal representative, to control access to personal health information. This means that families must act as the gatekeepers of any cross-sectoral data sharing.

Participants’ concerns thus reflect not only legal and ethical constraints but also deeper apprehensions about the potential misinterpretation or misuse of clinical information when transferred outside its original context. To mitigate these risks, some participants suggested sector-specific access protocols and stressed the importance of contextualizing shared observations to preserve the integrity and meaning of the information exchanged.

#### Family Engagement: From Genuine Partnership to Deeper Tensions in Power Dynamics

Most professionals identified the potential of patient portals to enhance family engagement in the rehabilitation process. Since family training and the codefinition of goals with parents significantly improve success in therapy [54], supporting rehabilitation continuity at home by involving parents is essential. Participants underscored the limitations of therapy time in transferring skills to the home environment, positioning portals as valuable tools for effective communication with parents. Furthermore, by promoting greater consistency, patient portals could help reduce family exhaustion, a critical issue given the challenges parents face in coordinating care [36,37]. Further, several professionals mentioned shared portals as a means to foster a genuine family partnership. Although patient- and family-centered approaches are frequently mentioned in pediatric rehabilitation, the concept of partnership remains less explored [55]. Partnership extends beyond a centered model, advocating for a more egalitarian relationship based on co-construction and shared decision-making [56,57]. Shared digital tools could reinforce this shift by enhancing transparency, offering parents access to rehabilitation goals and progress updates, and fostering their active engagement in the rehabilitation process [58]. Additionally, such tools could help professionals make their communication more accessible to children and families, thereby improving digital health literacy, which is a major determinant of health [59].

However, professionals’ ambivalences about the extent of parental access reveal deeper tensions between the principles of partnership and preserving professional autonomy. Some participants saw parents as coactors in the care process, but others feared that parental oversight could inhibit candid communication among professionals. These tensions may reflect different perspectives regarding family-centered care [60], especially as some families perceive their role as collaborative partners [40,41]. These power dynamics in therapist-parent relationships may further challenge the use of shared health portals by hindering shared decision-making [61].

Importantly, these tensions are further complicated by legal considerations. In France, health information may be shared between professionals only if the patient does not object [62], and, in the case of minors, legal representatives have the right to access their child’s health information and make decisions on their behalf [63]. This legal provision affirms the right of families to participate in their child’s care, but may conflict with professionals’ desires to preserve a confidential space for interprofessional collaboration. Designing patient portals in pediatric care must therefore navigate these complex dimensions, supporting meaningful family engagement, respecting professional communication needs, and ensuring their engagement while complying with legal requirements. These challenges reflect fundamentally different ethical stances on responsibility and access. As such, future implementations should consider flexible access models and co-designed governance frameworks to ensure shared ownership that honors legal and ethical responsibilities.

#### Children's Engagement: From Empowerment to Protective Gatekeeping

Professionals recognized that involving children in shared portals could promote key aspects of self-determination, such as autonomy, self-expression, motivation, and increased self-efficacy [64]. Features such as child-defined goals and progress graphs hold great potential to respond to these needs and to sustain motivation and long-term involvement, which is crucial in pediatric rehabilitation pathways that often extend over years [65].

However, participants also expressed multiple concerns about the risks of granting direct access to children. Concerns related to the age of consent echo the limited but growing literature on children’s cyber safety [66]. This vigilance may be justified given the dual vulnerability of minors with disabilities, and highlights the need for strategies that consider children’s evolving capacity to consent and support their gradual, developmentally appropriate access to such portals. Additional concerns were raised about increased screen time and the associated risks of excessive exposure in children [67]. However, the use of such a portal can be meaningfully distinguished from passive or recreational digital consumption, as it would be designed to involve adult guidance, whether by professionals during sessions or by parents, and to support goal-oriented, purposeful engagement with progressive access based on the child’s capacities [68].

At the same time, this ambivalence between the need to improve the involvement of children and the need to protect them reflects a deeper ethical conflict, aligning with studies on barriers to participation in pediatric rehabilitation, highlighting protective attitudes toward children among professionals [69]. Additionally, this protective instinct may unintentionally conflict with contemporary rights-based approaches for children and the right to receive transparent health information and to participate in decisions affecting their care [70]. Ultimately, the inclusion of children raised a central dilemma for professionals: should portals serve as tools for empowerment, or could they risk overexposing children? Designing portals for children should therefore not revolve around binary choices between access and restriction, but rather nuanced, developmentally appropriate solutions that support gradual access and informed participation. Addressing these questions is essential for ensuring ethical and age-appropriate design but also for advancing equity and agency in pediatric rehabilitation [71].

#### Beyond the Time Required to Use Portals, a Matter of Professional Beliefs About Their Coordination Role

Issues regarding time required for portal use are consistent with previous studies [20,21,51]. However, this study revealed that professionals’ willingness to adopt such tools goes beyond practical limitations. Their intention to use portals was closely linked to their belief that it could enhance their ability to deliver quality care. While some participants viewed portals as essential to improving coordination, others saw them as an additional burden. This ambivalence suggests a partial intention to adopt the tool, consistent with the Technology Acceptance Model, which posits that perceived usefulness influences user acceptance [72].

Beyond individual beliefs, this hesitancy also points to the need for targeted implementation strategies to support full adoption, in line with the Theoretical Domains Framework [73], which emphasizes the importance of addressing behavioral determinants of change. Participants’ concerns must therefore be taken seriously to ensure the successful integration of portals in pediatric rehabilitation. This includes addressing the broader transformations required: strengthening interprofessional and cross-sectoral collaboration, particularly with schools, and promoting patient- and family-partnered care through shared decision-making.

These findings echo the argument by Goodwin [74] that truly integrated care requires a dual disruptive innovation: not only the development of digital tools, but also a transformation in the way professionals work together and engage with children and families to coproduce care.

### Next Steps

The professionals’ perspectives will be combined with families’ and children’s perspectives and carried forward as inputs into the upcoming phases of the project. Focus groups with stakeholders will be conducted to align needs and concerns across groups [75] and to refine portal features through participatory design [27]. This pragmatic approach will ensure that professionals’ concerns are considered from the outset. Subsequent stages will involve defining implementation strategies with decision makers from health, education, and recreation settings, to support adoption in real-world settings, with particular attention to interoperability challenges with electronic health records. Ultimately, the portal will be developed in partnership with a technology company.

### Limitations

The sample was unbalanced, with more health care professionals from rehabilitation settings than from education, recreation, and social services. This is due to the central role of these professionals in rehabilitation and the recruitment primarily occurring through the FRISBEE [35] network. Nevertheless, the maximum variation sampling across profession, age, digital literacy, and working facility—combined with snowball sampling—enabled the collection of rich and nuanced insights into the phenomenon under study [37].

The individual interviews allowed us to explore a broad range of opportunities and concerns, which was relevant given the variability of rehabilitation settings in France. However, focus groups could have allowed participants to compare their viewpoints and generate additional ideas on the implementation strategies for portals, for example [76]. Regarding implementation strategies, it is important to note that the data collected here, although informative, is insufficient to draw conclusions on which specific strategies should be adopted for successful portal implementation [77]. This study is a preliminary step, and further research will be needed to identify the most effective strategies. Future work should incorporate solid theoretical frameworks in implementation science and involve key decision makers to understand the organizational factors influencing the implementation process [78].

The analysts were mainly health care professionals, sharing similar characteristics with some participants, which may have contributed to a greater emphasis on these perspectives. However, the broader team was multidisciplinary, and regular meetings were held for critical reflexive analysis. Moreover, the inclusion of parent partners’ perspectives contributed to a more comprehensive and in-depth analysis [79].

### Conclusions

This study provides valuable insights into how digital health portals could support integrated pediatric rehabilitation care, from the perspectives of professionals working with children with disabilities. Professionals emphasized not only the potential of these tools and key features to enhance care quality but also the ethical and legal challenges their use poses. Their ambivalence highlights the need for portals that facilitate communication while respecting professional roles, family engagement, and child participation. Developing such tools requires more than technical solutions—it calls for a rethinking of how professionals collaborate with children, families, and other professionals. Co-design with these stakeholders and decision makers will be essential to ensure relevance, usability, and real-world impact.

## Data Availability

The datasets generated or analyzed during this study are available from the corresponding author on reasonable request.

## Appendix

Multimedia Appendix 1 Interview guide.

Multimedia Appendix 2 COREQ checklist.

## Abbreviations

COREQ: Consolidated Criteria for Reporting Qualitative Research
FRISBEE: Fédération Médicale et Universitaire des SMR Pédiatriques de Bretagne Occidentale
