# Supplementary material for: Perspectives From Multidisciplinary Professionals in France on Shared Patient Portals for Integrated Pediatric Rehabilitation: Qualitative Study
Source: JMIR Rehabil Assist Technol. 2025 Oct 10;12:e73068. doi: 10.2196/73068 (PMC12552821; doi:10.2196/73068)
Supplement: Multimedia Appendix 1 [file rehab_v12i1e73068_app1.docx]

[Introduction and information about confidentiality]

***Part 1. Digital health technologies experiences, perceived usefulness, opportunities, and concerns about shared patient portals and features desired***

1. **Have you ever used digital health technologies to help coordinate care or monitor your patients in pediatric rehabilitation?**
   1. [prompt] if yes: Which application? Can you tell me about it? What did you like/dislike?
   2. [prompt] if no: Would you be interested in using it? Why?
2. **What do you think of shared patient portals to facilitate care coordination and follow-up for children undergoing rehabilitation?**
   1. [prompt] What opportunities do you perceive? For children? For families? For you? For quality of care?
3. **What information would you like to exchange through shared portals?**
   1. [prompt] What information should be exchanged to improve the child's rehabilitation pathway?
   2. [prompt] Do you have examples of children you’re following to illustrate the need of shared patient portals?
4. **With whom would you like to share information through a shared portal?**
   1. [prompt] How do you perceive the role of families in portals?
   2. [prompt] How do you perceive the role of patients in portals? At what age do you think children should have access to this type of portal?
   3. [prompt] How do you perceive your role in portals?
   4. [prompt] Which other professionals should have access to such portals?
5. **What features should be developed to meet the needs you’ve outlined?**
   1. [prompt] Concretely, how do you imagine that shared patient portals could meet the challenge/need you describe? Through which feature?
6. **What are your concerns about a shared patient portal?**
   1. [prompt] What are your fears when you think of the practical use of shared patient portals in pediatric rehabilitation? Why? Do you have any examples?

***Part 2. Demonstration of the Deneo patient portal for adults, other desired features, and strategies to facilitate the use of shared patient portals in practice***

[short demonstration of features of an existing health portal developed for adult rehabilitation (https://deneo.app/): 1) sharing of reports, images, photos and videos between patients and professionals, 2) a database of validated rehabilitation measurement scales and questionnaires, 3) monitoring patient progress using graphs, and 4) prescription of self-rehabilitation exercises]

1. **What do you think of this patient portal?**
   1. [prompt] What do you like/dislike?
   2. [prompt] What do you think of the proposed features? Do you have any other ideas for features?
2. **In practice, how do you see yourself using a digital health patient portal?**
   1. [prompt] How could the tool fit into your practice?
   2. [prompt] What would motivate you to use it? What would increase your motivation to use it?
3. **What strategies could be implemented to facilitate the use and uptake of shared portals in pediatric rehabilitation?**
   1. [prompt] Do you have other recommendations regarding the implementation?
   2. [prompt] Is there anything else that you would like to tell us about digital health technologies in pediatric rehabilitation?
   3. [prompt] Do you have a word or a sentence to end this interview with?

[Conclusion and acknowledgements]
